# Effects of Primary Varicocele and Related Surgery in Male Infertility: A Meta-Analysis

**DOI:** 10.3389/fsurg.2020.586153

**Published:** 2020-10-30

**Authors:** Xinkun Wang, Tong Chen, Junfeng Qiu, Hongwei Wu, Xuehua Chen, Xujun Xuan

**Affiliations:** ^1^Department of Andrology, The Seventh Affiliated Hospital, Sun Yat-sen University, Shenzhen, China; ^2^Department of Pediatric Surgery, Shanghai Children's Hospital, Shanghai Jiao Tong University, Shanghai, China; ^3^The Key Laboratory for Reproductive Endocrinology of Ministry of Education, National Research Center for Assisted Reproductive Technology and Reproductive Genetics, Center for Reproductive Medicine, Shandong University, Jinan, China

**Keywords:** natural conception rate, operation treatment, varicocele, surgery, infertility

## Abstract

**Purpose:** To investigate the effect of primary varicocele and related surgery in male infertility through meta-analysis.

**Methods:** A systematic search of the literature was conducted using the Medline, Embase, Cochrane, and CNKI databases. The search was up to September 2019. Article selection proceeded according to the search strategy based on Preferred Reporting Items for Systematic Reviews and Meta-analyses criteria. Data were analyzed using RevMan 5.2. A random-effects model was used to calculate the overall combined risk estimates.

**Results:** After screening 687 articles, 4 randomized controlled trials with 349 patients were included. One hundred seventy two patients were addressed in embolization/ligation, with 177 patient's observation treatment. The number of spontaneous pregnancies in the two groups was 41 and 40, respectively. There was no significant difference in pregnancy rate between the operation group and the control group. RR = 1.05 [0.72, 1.54].

**Conclusion:** There is not enough evidence to explain the surgical treatment of varicocele can improve the natural fertility of the infertile couples, and there is still a need for most of prospective randomized controlled trials to verify the efficacy of varicocele surgery for treating of male infertility. We do not deny the importance of this operation, we just want to call on everyone to strictly grasp the indications of the operation, avoid ineffective medical expenses, and avoid unnecessary pain to patients.

## Introduction

Varicocele (VC) is defined as a vascular disease, which refers to the abnormal expansion, elongation, and tortuosity of the venous plexus in the spermatic cord. It was first treated as a disease in 1829 and is considered to be common cause of male infertility. For over half a century, high spermatic vein ligation, a routine surgical method for the treatment of varicocele, has been applied as a routine surgical method in infertile men.

In previous studies, varicocele was mostly regarded as one common cause of male infertility (1–3). In men, the incidence of varicocele is about 15% (1–3). Among men with primary infertility, the incidence of varicocele accounts for about 35%, and secondary infertility can amount to 80% (1). At present, most scholars have suggested that varicocele may be related to abnormal semen parameters in men and may be one of the causes of male infertility. The adverse effects of varicocele on fertility may be related to the following factors: increased testicular temperature, hypoxia, hormonal disorders, insufficient testicular perfusion, toxic metabolites reflux, oxidative stress, and so on (2–4). However, the mechanism of varicocele influence on male infertility has not been fully elucidated, and the mechanism of fertility recovery by varicocele surgery remains unclear.

At present, there are many surgical methods for varicocele, including traditional high ligation of the spermatic vein, modified Palomo varicocele ligation, laparoscopic high spermatic vein ligature, microscopic spermatic vein ligature, vascular embolism, sclerosing agent, etc. It is mostly believed that surgery is the most effective way to treat infertility caused by varicocele. Despite this, it is not rare to see that many men with varicocele have normal semen quality and normal fertility, and some patients with varicocele do not recover after surgery. Therefore, some researchers believe the value of varicocele treatment is questionable (5, 6). The National Collaborating Center for Women's and Children's Health: “Surgery for patients with varicocele should not be provided as a form of fertility treatment because it does not increase the pregnancy rate” (7). In guidelines raised by European Association of Urology, pregnancy treatment for varicocele remains controversial (8).

By reviewing the literature, it was found that most of the studies on the effect of varicocele surgery are based on semen parameters as research indicators, and relatively few studies have used natural fertility rates as research indicators. In addition, most of the studies that use natural conception as the research index have not ruled out the influence of related reason. This meta-analysis attempted to conduct objective literature review and data analysis on whether the surgical treatment of varicocele increased the spontaneous pregnancy rate of infertile couples.

## Methods

### Study Retrieval

We used electronic retrieval and manual retrieval to conduct preliminary literature inspection. Electronic retrieval databases include PubMed (Building Database-2019.9.1), Chinese Journal Full-text Database (CNKI: Building Database-2019.9.1), Embase Database (Building Database-2019.9.1), Cochrane Database (Building Database-2019.9.1) and major professional magazines. The search terms were Varicocele, surgery, infertility, and semen. Randomized controlled studies with pregnancy as the main outcome was included, and the study was performed in the surgical treatment group (Interventional) and untreated groups. The data was independently extracted by two authors.

### Inclusion Criterion

Each study was reviewed independently by two investigators. Eligibility for study selection was defined by the patient population, intervention or exposure, comparator, outcome, and study design (PICOS). A record was considered relevant to this review if it assessed men with Varicocele and Suffer from infertility (P); the use of Surgery (I); compared the Surgery with observation treatment control group (C); and patient outcomes of natural fertility (O). The selection criterion included: Regular sexual life of both spouses, and without contraception, factors leading to female infertility were excluded, and unfertile for 1 year. The man was diagnosed with unilateral or bilateral clinically accessible varicocele by color Doppler ultrasound or thermal imaging. Semen analysis results routinely indicate the presence or absence of parameter abnormalities.

### Data Extraction

Data of each study was extracted independently by two investigators. In event of any disagreement, rechecking, and discussion were conducted under the guidance of the corresponding author. The demographic and clinical characteristics of included studies were documented. The following outcomes were extracted for analysis: (1) Number of people included; (2) Number of people follow-ups; (3) Follow-up time; (4) Whether to exclude the man's other diseases (such as epididymitis, abnormal hormone levels, etc.); (5) Whether to exclude the woman's disease; (6) Man's age; (7) Woman's age; (8) Surgical approach; (9) Number of pregnant; (10) Natural conception rate.

### Statistical Analysis

Statistical analysis was performed in accordance with the guidelines for statistical analysis developed by the Cochrane Collaboration. Heterogeneity (variations) between the results of different studies was examined by inspecting the scatter in the data points on the graphs and the overlap in their confidence intervals and, more formally. The outcomes were pooled statistically. Results for each study were expressed as a relative risks (RR) with 95% confidence intervals (CI) and combined for meta-analysis with RevMan 5.2.

## Results

### Evaluation of Included Studies

**Figure 1:**
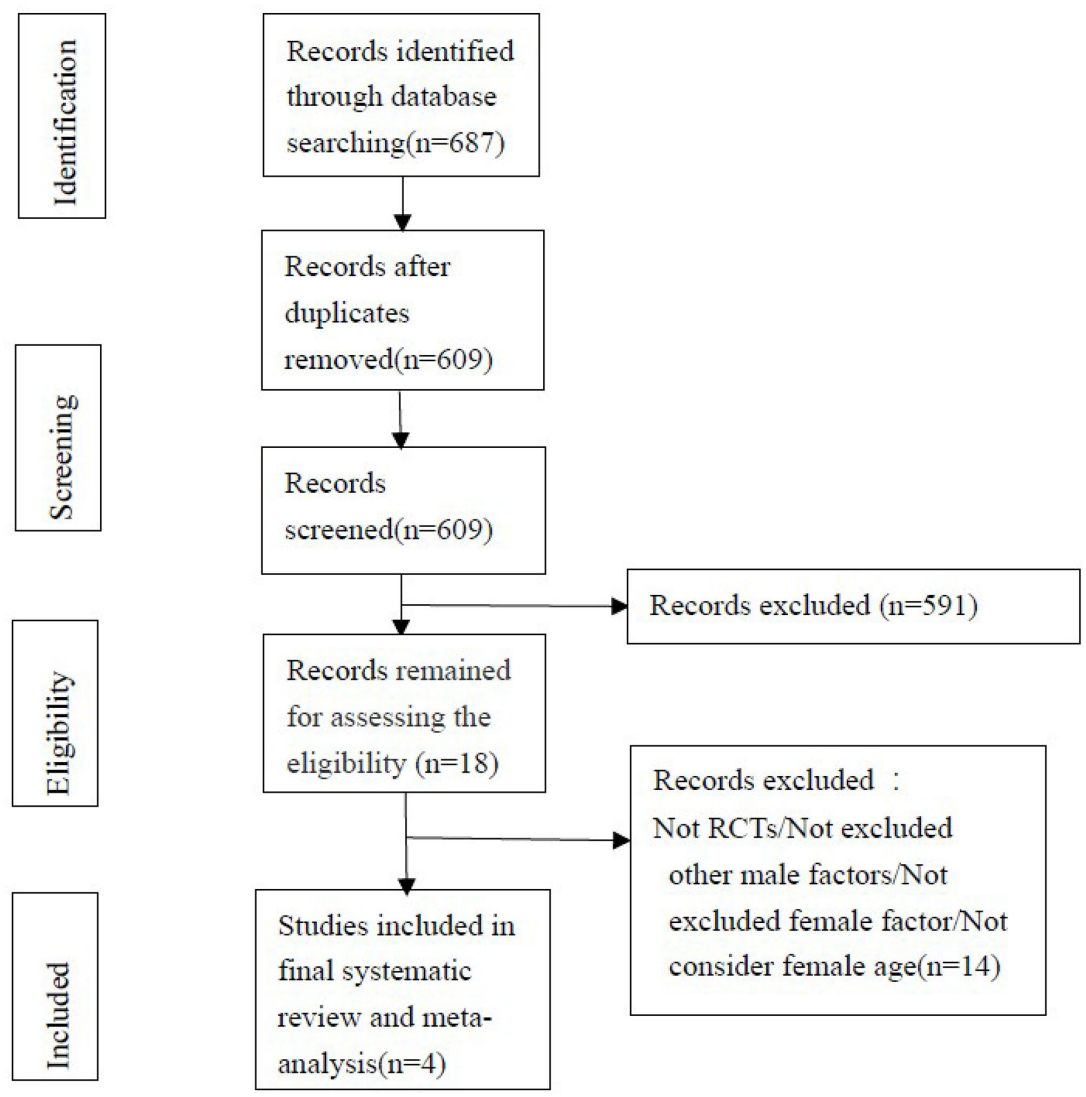
Flow diagram outlining the search strategy and study selection process for this review. RCTs, randomized controlled trials.

The search strategy is shown in Figure 1. We retrieved 189 articles from the Embase database, 96 articles from the PubMed database, 317 articles from CNKI, and 85 articles from Cochrane. A total of 687 eligible studies were retrieved in our database search.

By excluding conference papers, reviews, retrospective studies, and other factors that cause infertility in men, such as: urogenital surgery, epididymitis, cryptorchidism, mumps, orchitis, gonadal dysplasia, abnormal sex hormones, etc, serious systemic diseases and endocrine diseases; use of cytotoxic drugs, Immunosuppressants, anticonvulsants, azoospermia, or semen volume <1 ml; and the spouse has factors that cause infertility, such as blocked fallopian tubes, endometriosis, persistent amenorrhea, older (45 years old) Above) etc. There are 18 papers selected initially. One of them is a continuation of a previously published literature (9, 10).

Of the 18 articles, 9 of the randomized controlled methods were used; 15 of the other causes of infertility were excluded from the exclusion of the male; 14 of the female infertility factors were excluded; 9 of the women undefined age were mentioned; and 8 of the pre-operative level examinations were completed. Unfortunately, in the above 15 articles which has excluded male factors, although the “excluding Other causes of infertility due to male,” there is no specific explanation that the AZF deletion, the CFTR mutation, and the problem of anti-sperm antibody is excluded.

**Figure 2:**
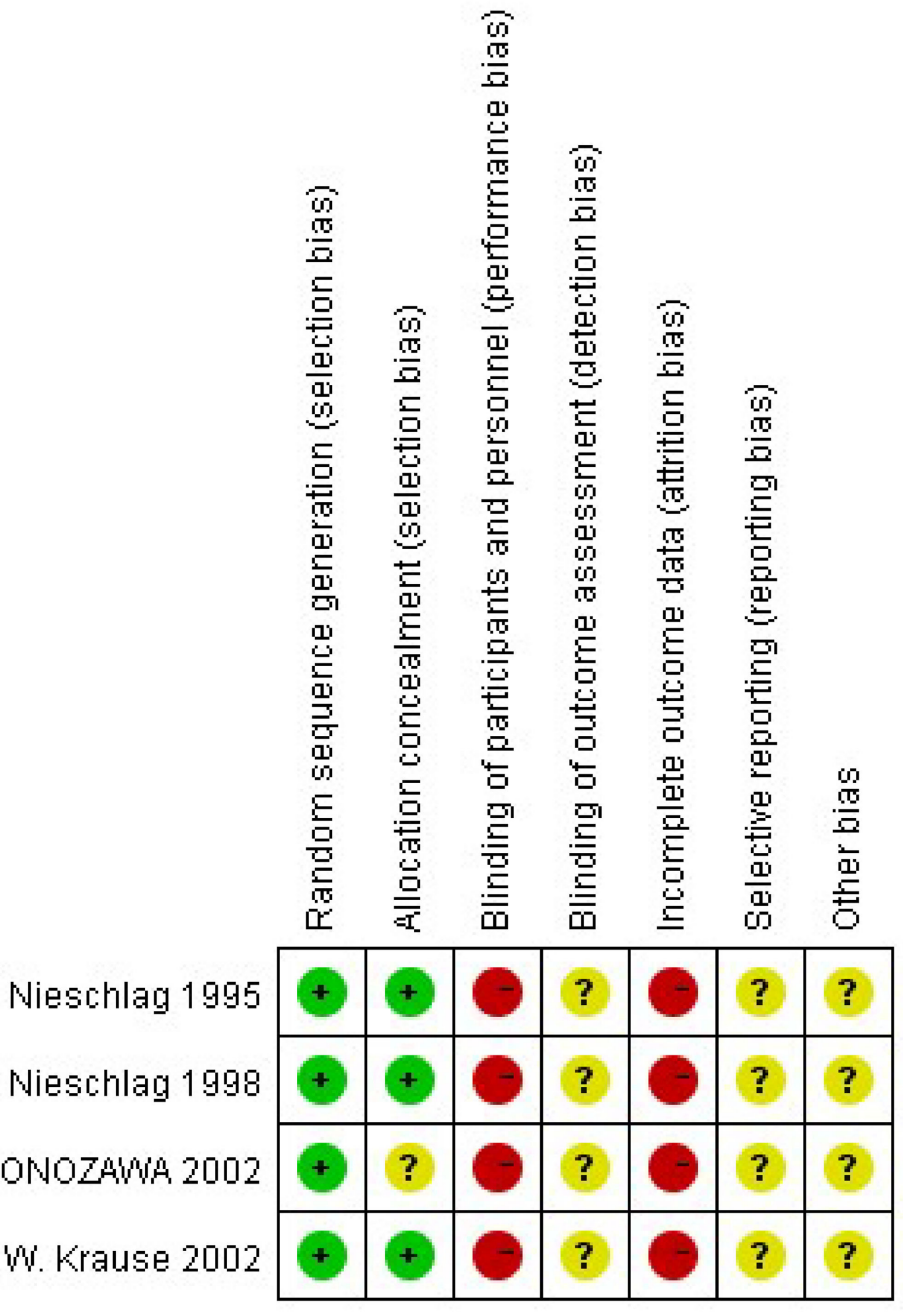
The methodological quality of each study by four-item scale of risk of bias.

Four items were included in this study (9–12). The risks and biases of these four studies are analyzed, and the results are shown in the Figure 2. The heterogeneity of these four articles was analyzed, and it was found the homogeneity was poor (*P* ≤ 0.93), so the Randomized effects model was used. Since there are only four included studies, no publication bias analysis was performed. Finally, 349 patients were included, including 172 cases in the operation group and 177 cases in the observation group. Three methods, high ligation, embolization, and sclerotherapy, are mainly mentioned. Nieschlag (9) mentioned that a previous study has demonstrated that surgical ligation and radiological embolization are equally effective in terms of pregnancies following treatment. Similarly, some studies have shown that a similar efficacy of sclerotherapy and surgical treatment with respect to sperm parameters (6, 13). The number of spontaneous pregnancies in the two groups was 41 and 40, respectively. There was no significant difference in pregnancy rate between the operation group and the control group. RR = 1.05 [0.72, 1.54] (Table 1).

**Table 1:** Comparison of conception status between operation group and control group.

	**Operation group**		**Control group**			**Risk ratio**	**Risk ratio**
**Study or subgroup**	**Events**	**Total**	**Events**	**Total**	**Weight %**	**M–H random 95% Cl**	**M–H Random 95% Cl**
Nieschlag et al. (9)	12	47	13	48	30.1	0.9427 [0.4806, 1.8491]	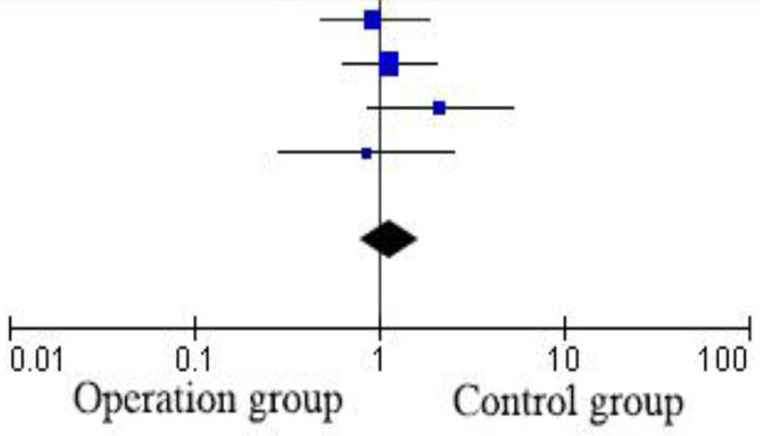
Nieschlag et al. (10)	18	62	16	63	41.4	1.1431 [0.6433, 2.0314]	
Onozawa et al. (12)	6	10	5	18	16.9	2.1600 [0.8777, 5.3156]	
Krause et al. (11)	5	32	6	33	11.7	0.8594 [0.2911, 2.5372]	
Total (95% Cl)		151		162	100.0	1.1615 [0.8025, 1.6811]	
Total events	41		40				

*Heterogeneity: Tau^2^ = 0.00; Chi^2^ = 2.52, df = 3 (P = 0.47); I^2^ = 0%.*

*Test for overall effect: Z = 0.79 (P = 0.43)*.

## Discussion

**Figure 3:**
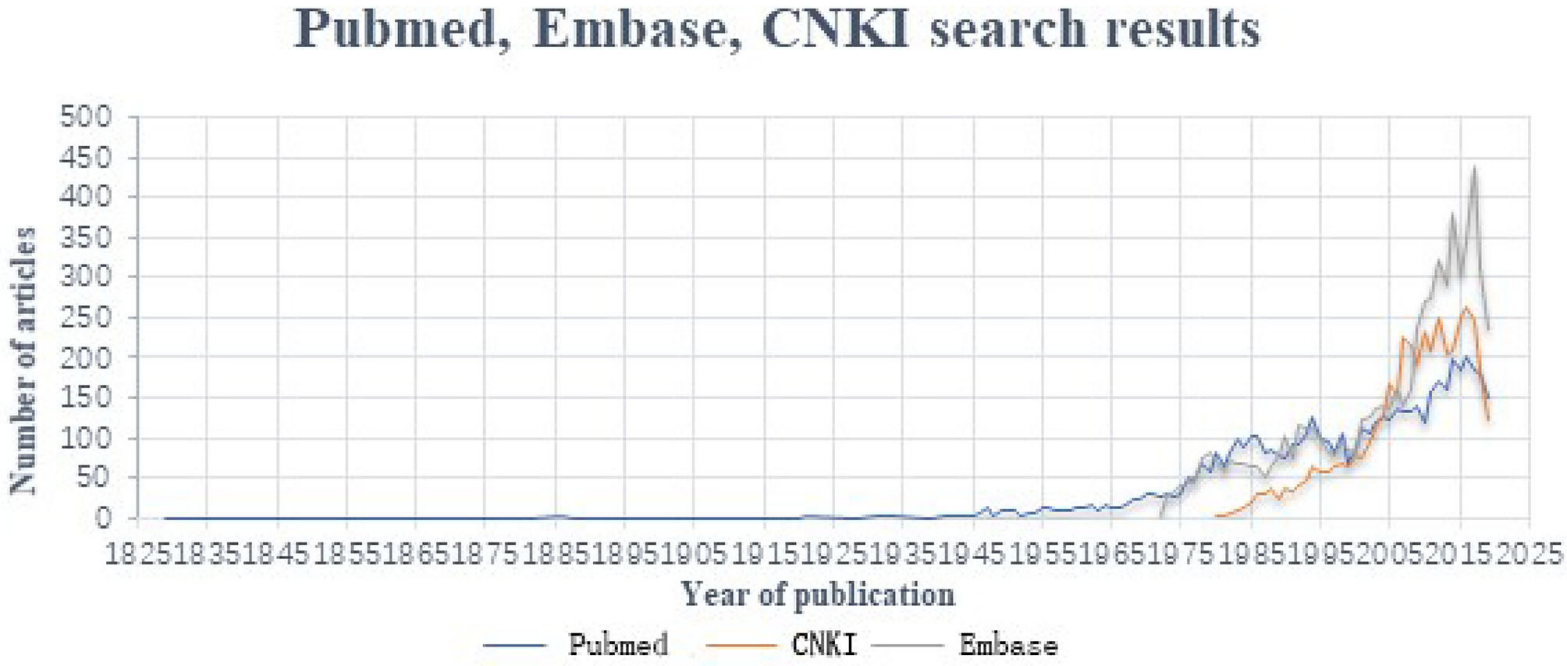
The number of documents in each database changes with time.

In the current study, Varicocele was applied as the key word to search the major databases. The retrieval time is from the time database was created to 2019.9, Embase Database retrieves 6,574, PubMed retrieves 5,424, and CNKI retrieves 4,319 articles. The number of documents in each database is as follows (Figure 3).

As shown above, since 2000, the literature on varicocele has increased sharply. There are 4,608 articles on Embase, 2,924 articles on PubMed and 3,608 articles on the knowledge net. Specifically, a large part of the literature searched comes from Chinese research institutions.

Varicocele is a common clinical manifestation of male spermatic cord, which was first treated in 1829 (14). Epidemiologically, about 15% of men suffer from this disease, the incidence of varicocele in primary infertile patients is about 35%, and the incidence of secondary sterility is as high as 70–85% (15–17). Interestingly, about 80% of all patients with varicocele are fertile (18). Is it necessary to perform surgical intervention to treat varicocele?

Nowadays, many studies have confirmed that varicocele can decrease semen quality. However, there is no mechanism fully explaining the relationship between varicocele and infertility. Tortuous internal spermatic veins cause poor blood reflux, resulting in elevated scrotal temperature, hypoxia, hormone disorder, insufficient testicular perfusion, toxic metabolites reflux, oxidative stress, and so on, which are the probable causes of varicocele-mediated sterility (2–4). In view of the above reasons, some scholars have proposed that the removal of disordered and tortuous blood vessels can correct the deterioration of semen quality and male sterility attributable to varicocele, at least theoretically. Therefore, in the past 20 years, the surgical treatment of varicocele has been updated frequently and has a wide range of specific surgical methods, such as: high spermatic vein ligature, modified Palomo varicocele ligation, laparoscopy internal spermatic vein ligature, microscopic internal spermatic vein ligature, vascular embolism, sclerosis agent, and so on. With the education of health knowledge, the improvement of people undefined health consciousness and the continuous progress of diagnosis and treatment, the diagnosis rate of varicocele is increasing. Just as the literature of varicocele “blowout,” there is more and more surgical methods of varicocele. Among them, some doctors are not certain about the indication of varicocele surgery, and even expand the surgical indication, resulting in one condition that some patients who did not need surgical treatment also underwent varicocele surgery. What undefined more, some hospitals regard VC as an important cause of male sterility and operate, which expands the indication of operation and becomes a varicocele ward. This kind of “excessive medical treatment” not only brings physical injury and economic burden to patients, but also aggravates the tension of social healing resources and the waste of medical insurance resources.

In past studies, although some scholars have explored the impact of varicocele surgery on infertility through meta-analysis, they did not consider the comprehensive reasons of both men and women. In our research, we passed strict screening. Six hundred eighty seven eligible studies were retrieved in our database search. We found that in the studies using the natural conception rate as the result, a large part did not exclude the influence of male hormone levels and fertility-related biochemical signs and the effect of the age of the spouse. Studies that exclude the genetic level (such as AZF deletion. CFTR mutation) and the immune level (such as anti-sperm antibodies) are rare. After screening, 4 eligible studies were included for analysis, containing 172 patients receiving surgery and 177 patients receiving observation treatment. The demographic and clinical characteristics of these studies are listed in Table 2. The number of spontaneous pregnancies in the two groups was 41 and 40, respectively. There was no significant difference in pregnancy rates between the operation group and the control group, which were 23.84 and 22.69% respectively. RR = 1.05 [0.72, 1.54]. Therefore, the effectiveness of surgery for varicocele patients with infertility remains to be discussed.

**Table 2:** Data extraction from four studies.

	**Krause et al. (11)**	**Nieschlag et al. (9)**	**Nieschlaget al. (10)**	**Onozawa et al. (12)**
Number of people included	67	95	125	Total:64 Operation: 31 Observation: 33
Number of people follow—ups	Total: 65 Operation: 32 Observation: 33	Surgery: 23 Embolism: 24 Observation: 48	Surgery: 30 Embolismt: 32\Observation: 63	Surgery: 10 Observation: 18
Follow—up time	1 year	I year	I year	76.2 months
Whether to exclude the man's other diseases (such as epididymitis abnormal hormone levels, etc.)	Excluded	Excluded	Excluded	Excluded
Whether to excluded the woman's disease	Excluded	Excluded	Excluded	Excluded
Man's age	32.2 ± 5.8 (65)	Surge1y: 32.7 ± 0.8 Embolism: 32.6 ± 0.9 Observation: 32.8 ± 0.5	(Surgery + embolization): 33.1 ± 0.4 Observation: 32.8 ± 0.5	Operation: 34.4 ± 5.2 Observation: 35.1 ± 5.4
lVoman's age	29.7 ± 4.8 (56)	Embolism: 30.1 ± 0.7 Operation: 31.0 ± 0.8 Observation: 30.4 ± 0.5 Pregnancy: 28.4 ± 0.65 Not pregnant: 31.2 ± 0.40	(Surgery + embolization): 30.4 ± 0.4 Observation: 30.5 ± 0.5 Pregnancy: 28.8 ± 0.6 Not pregnant: 31.2 ± 0.3	Operation: 31.3 ± 3 3 Observation: 30.7 ± 5.0
Number of pregnant	Surgery: 5 Observation: 6	Surgery: 12 Observation: 13	(Surgery + embolization): 18 Observation: 16	Surgery: 6 Observation: 5
Natural conception rate	Surgery: 15.63% Observation: 18.18%	Surgery: 25.5% Observation: 27.1%	(Surgery + embolization): 29.03% Observation: 25.40%	Surgery: 60% Observation: 28%

Fertility depends on various, and treat male sterility is also multifaceted. It is, therefore, difficult to get valuable results without strictly controlling other variables and simply discussing the effects of surgery. According to our clinical experience, treatment of male infertile varicocele is rarely done only by operation without other treatments. Antibiotics, vitamin C, vitamin E, Levocarnitine and other antioxidants, seven-leaf saponins, and proprietary Chinese patent drugs will also be used for auxiliary treatment before and after operation, which will also improve semen quality in a short period of time (for example, 1–3 months). This is therefore difficult to explain whether improve semen quality after operation is the effect of surgery or related auxiliary medication.

We do not deny the importance and significance of surgery. We want to call on everyone to strictly grasp the indications for surgery, and to avoid surgery for patients who can be conceived through non-surgical treatment, and not to “only operate,” so as to avoid ineffective medical expenses, and avoid the suffering of patients. For infertile men with Varicocele, if the semen test results are normal, surgery can be temporarily ignored, follow up observation, and attention to look for other infertility factors, especially to evaluate of the fertility of the spouse. For Varicocele men with abnormal semen quality, Varicocele may not be the only cause or the main cause of infertility. patients may have other diseases or abnormalities that affect their fertility, which can be treated with conservative drugs. And attention to look for other infertility factors, Other abnormalities have been ruled out, and the semen quality is consistent with Varicocele, at which point the Varicocele is suspected of affecting male fertility. Active intervention at this point is more likely to yield satisfactory results.

The causes of infertility are complex. Varicocele may only be one of the causes, and the author believes that it is only a disadvantageous factor and does not play a decisive role. The prevalence of varicocele is mainly evaluated in young adults. From the point of view of human and biological evolution, if varicocele is closely related to fertility and plays a key role, then in the early stage of human origin and primitive society, human life expectancy is short, <20 years old. People who should be in peak fertility but have varicocele may be eliminated by natural selection because of the decline in fertility! Moreover, if varicocele is closely related to fertility, the more severe varicocele is, the worse the semen quality is and the lower the fertility is. However, some studies also found that there was no significant differences in semen analysis among VC patients with different severity (19), but the effect of testicular volume atrophy on semen quality was more obvious (20). However, the relationship between varicocele and testicular volume still has yet to be studied.

In fact, many scholars are also aware of these problems, and the Evers Johannes and Collins (21) have raised questions about the effect of treating varicocele to treat male infertility in 2004. A number of scholars also conducted a prospective randomized controlled study (22–25), but the methods were not satisfactory, and none of the studies completely excluded the interfering factors (e.g., hormone levels, gene levels, immune effects, spouse age, etc.). From the present study, semen quality after varicocele surgery is improved to a certain extent, but the rate of natural pregnancy is not much improved. The reason may be the “fertility window.” The fertility of women declines with age, and the “fertility window” is much narrower than that of men. Only when the couple undefined “fertility window” is combined, it is possible to achieve the purpose for the evaluation of fertility. Therefore, the treatment of male infertility related to varicocele should not only strictly grasp the indications, but also cooperate with the fertility status and fertility of the spouse.

## Conclusion

There is no enough evidence to explain the surgical treatment of varicocele can improve the natural fertility of the infertile couples, and there is still a need for most of prospective randomized controlled trials to verify the efficacy of varicocele surgery for treating of male infertility. We do not deny the importance of this operation, we just want to call on everyone to strictly grasp the indications of the operation, avoid ineffective medical expenses, and avoid unnecessary pain to patients.

## Data Availability Statement

All datasets generated for this study are included in the article/supplementary material.

## Author Contributions

XW and TC: study design. TC, JQ, HW, and XC: collection and analysis of data. XW, TC, and XX: manuscript writing. All authors contributed to the article and approved the submitted version.

## Conflict of Interest

The authors declare that the research was conducted in the absence of any commercial or financial relationships that could be construed as a potential conflict of interest.
